# Physicians’ Reports of Births, &c.

**Published:** 1880-08

**Authors:** 


Physicians' Reports of Births, &c.—We are informed by the Secretary of the City Health Department, that there is great remissness and lack of attention on the part of our physicians, or at least, some of them, in obeying the ordinance requiring the registration of all births occurring in their practice, within the limits of the city of Baltimore. This should not be the case. So important are the statistics of births and deaths, at the present day, to the well being of a city hygienically, and otherwise, that delinquents should be held to strict accountability in all cases when they fail to report, either, as they may occur in their practice. The law should be made *to compel* physicians to report all births and deaths, with their dates, sex, etc., as they take place over *their full names* and address, within twenty-four hours after their occurrence ; and these should be published weekly *with the name* of the attending physician. This would avoid all confusion and prevent mistakes that would be likely to occur otherwise. All will be ready to admit that statistics on these points, to be of any present or future value, should be accurate in every possible respect; and this desirable end can only be satisfactorily reached by the method mentioned above. A physician unwilling to aid in these vital statistics, should not be permitted to make his living in a community whose best interests he so completely ignores.

